# Madelung’s disease: A spot diagnosis

**DOI:** 10.4103/0970-0358.73472

**Published:** 2010

**Authors:** Noushif Medappil, T. A. Vasu

**Affiliations:** Department of General Surgery, Thiruvananthapuram Medical College, Kerala University, Thiruvananthapuram - 695 011, Kerala, India

Sir,

We wish to bring to notice of the readers a case of Madelung’s disease, characterised by doughy, non tender, subcutaneous, compartmentalised and unencapsulated adipose tissue masses mainly around the neck and upper trunk. The peculiar appearance of the patient usually renders a spot diagnosis of this benign disease.

A 59-year-old male presented with progressive and disfiguring swellings around the neck and shoulders of 3 years duration [Figures 1–3]. His past history was insignificant except for chronic alcoholism. The swellings were subcutaneous, doughy and non tender. His face, hips and legs remained unaffected. Biochemical parameters were within normal limits. Sonology of neck showed unencapsulated fat deposits. Fine needle aspiration from the swelling was suggestive of lipomatous lesion. We proceeded with lipectomy of neck for cosmetic reasons and the post operative period was uneventful.

**Figure 1:**
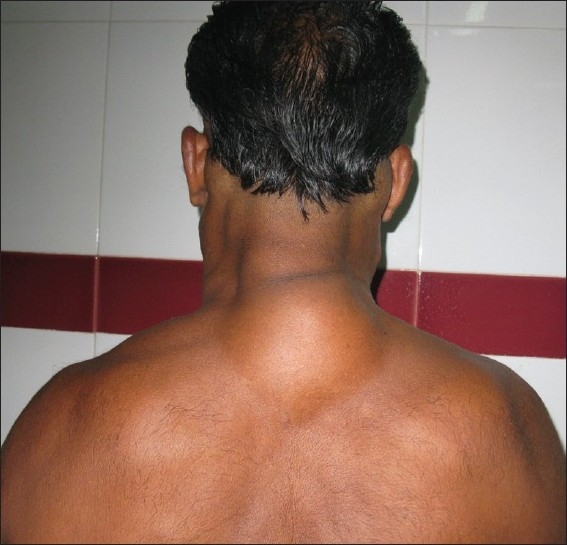
Buffalo hump

**Figure 2:**
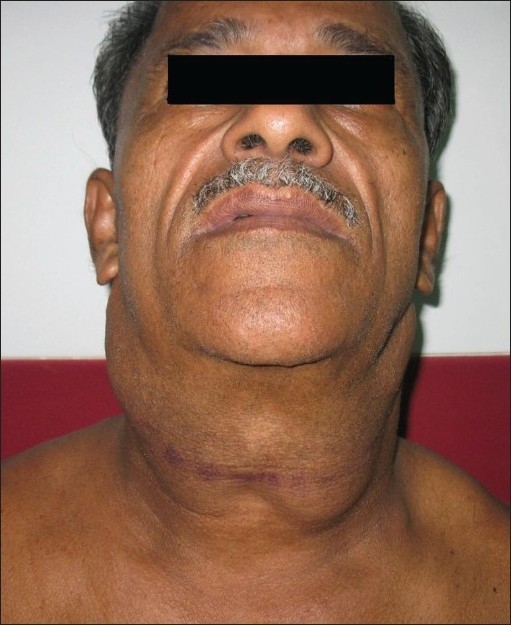
Horse collar

**Figure 3:**
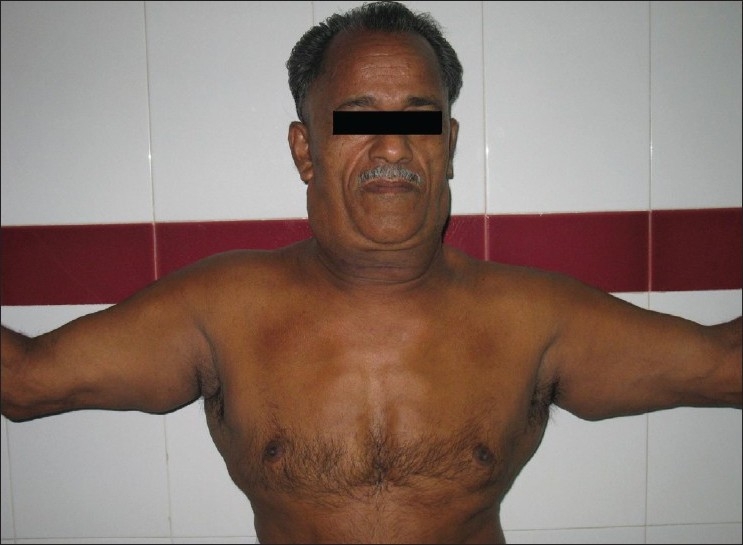
Pseudoathletic appearance

Madelung’s disease (multiple symmetric lipomatosis, benign symmetric lipomatosis, cephalothoracic lipodystrophy, Launois Bensaude syndrome), first reported in 1846 by Brodie, was called fat neck (Fetthals) by Madelung in 1888. Similar phenotype has been depicted in the carvings of Queen of Punt (near Egypt) who died nearly 34 centuries ago. It is a rare disease of unknown aetiology seen in adult males in the age group of 30–60 years. This disorder is more frequent in the Mediterranean area and more than 200 cases have been published in the literature of which 90% of the patients had history of alcoholism. The disease has also been reported in non alcoholics and females.[1] Association of symmetrical lipomatosis with diabetes mellitus, hyperlipoproteinemia, hypothyroidism, gout and systemic mitochondrial diseases has been described in literature.[1,2] It is often dismissed as simple obesity due to the absolute symmetry in the subcutaneous deposition of unencapsulated fatty masses. Pathogenesis of this disease is suspected to be a defect in the adrenergic stimulated lipolysis that results in massive accumulation of lipomatous tissue. Another theory on accumulation of embryological brown fat has also been proposed.[1,3] The symptoms of the patient are primarily due to the cosmetic disfigurement. Fat deposition in the parotid region (hamster cheeks), cervical region (horse collar), posterior neck (buffalo hump) and upper trunk gives the patient a “pseudoathletic appearance” that resembles the Italic statue of 6th century BC “Capestrano Warrior”, discovered in Abruzzi (Italy).[1,3] Some patients also present with peripheral neuropathy, autonomic nervous system manifestations and symptoms due to compression of the airway, oesophagus, carotids or venacava. The steady increase in weight, despite attempts at dieting, often leads to the judgment of “a non compliant patient.” There are two phenotypical variants of the disease, Types 1 and 2. Type 1 is common in males with protruding masses around the neck and upper thorax with relative sparing of the trunk and extremities. Type 2 is seen in females with predominant deposition over the proximal part of extremities and trunk resembling obesity.[1] Till date, there has only been one reported case of malignant transformation in benign symmetrical lipomatosis. An association of the disease with malignancies of the aerodigestive tract has been described by some authors.[4]

Workup of the disease includes clinical examination, sonography, computerised tomography and fine needle aspiration cytology. The important differential diagnosis includes liposarcoma, multiple familial lipomatosis, Dercum’s disease, neurofibroma, drug-induced lipomatosis (steroidal and antiretroviral drugs), angiolipoma and hibernoma. Alcohol abstinence prevents progression of the disease.[1] Surgical treatment includes lipectomy and liposuction.[1] The use of ultrasound assisted liposuction has also been reported.[5] Surgical intervention is complicated by the increased vascularity and the tendency of non encapsulated masses to invade the tissues around them. The disease almost never undergoes spontaneous degeneration and has a high propensity to recur.[5]

Our patient had none of the associated diseases except for alcoholism. The typical appearance is valuable in the spot diagnosis, which is stressed in this article.

## References

[1] Suresh Chandran CJ, Godge YR, Oak PJ, Ravat SH (2009). Madelung’s disease with myopathy. Ann Indian Acad Neurol.

[2] Becker-Wegerich P, Steuber M, Olbrisch R, Ruzicka T, Auburger G, Hofhaus G (1998). Defects of mitochondrial respiratory chain in multiple symmetric lipomatosis. Arch Dermatol Res.

[3] Josephson GD, Sclafani AP, Stern J (1996). Benign symmetric lipomatosis (Madelung’s disease). Otolaryngol Head Neck Surg.

[4] Guastella C, Borsi C, Gibelli S, Della Berta LG (2002). Madelung’s lipomatosis associated with head and neck malignant neoplasia: a study of 2 cases. Otolaryngol Head Neck Surg.

[5] Faga A, Valdatta LA, Thione A, Buoro M (2001). Ultrasound-assisted liposuction for the palliative treatment of Madelung’s disease: a case report. Aesthetic Plast Surg.

